# Cancer-Derived Exosomes: A Cross-Cancer Comparative Analysis of Exosomal Proteins and MicroRNAs

**DOI:** 10.3390/ijms27157057

**Published:** 2026-08-06

**Authors:** Jong Hyun Kim

**Affiliations:** Department of Biochemistry, School of Medicine, Daegu Catholic University, Daegu 42472, Republic of Korea; kimjohn@cu.ac.kr; Tel.: +82-53-650-4061

**Keywords:** exosomes, cancer biomarkers, microRNA, proteomics, liquid biopsy, tumor microenvironment, precision oncology

## Abstract

Exosomes are small extracellular vesicles that mediate intercellular communication and, in cancer, carry cargo that both reflects the donor tumor cell and influences recipient cells within local and distant microenvironments. Exosomal proteins and microRNAs have been reported individually across many cancer types, but rarely compared on a common basis; in this review, previously reported molecules from eight cancer categories—blood, breast, colon, kidney, liver, lung, prostate, and stomach—were compiled from curated repositories and re-analyzed within a single functional framework. In total, 3643 exosomal proteins (523 hematologic, 3120 solid-tumor) and 627,225 miRNA–target pairs, derived from 350 unique microRNAs, were organized using Gene Ontology, KEGG, and PANTHER annotation. Across cancers, proteins converged on a reproducible core—signaling, transport, cytoskeletal organization, and extracellular interaction—dominated by binding, catalytic, and transporter functions localized to membrane, vesicle, and extracellular compartments. Comparisons between hematologic and solid malignancies revealed both shared cancer-associated functions and context-dependent patterns linked to tissue origin and disease ecology. Together, these findings indicate that integrated protein-and-microRNA profiling offers a useful framework for understanding tumor communication, refining cancer classification, and advancing biomarker discovery, while underscoring that harmonized workflows, independent validation, and mechanistic follow-up remain necessary before descriptive enrichment outputs can support clinically robust applications.

## 1. Introduction

Exosomes are nanoscale extracellular vesicles, typically 30–150 nm in diameter, that are generated through the endosomal pathway and released by most mammalian cell types. Their biological importance resides in the fact that they transport complex molecular cargo, including membrane proteins, cytosolic proteins, lipids, messenger RNAs, microRNAs, and other noncoding RNAs, from donor cells to recipient cells. This capacity enables them to function as mobile information carriers rather than inert cellular debris. In cancer biology, this property is especially relevant because exosomal cargo may reflect the physiological state, genetic alterations, stress responses, and microenvironmental adaptations of the parental tumor cell while simultaneously modifying the behavior of surrounding stromal and immune cells. The dual role of exosomes as both readouts and effectors of tumor biology has positioned them at the center of contemporary interest in cancer biomarker discovery, intercellular signaling, and precision medicine [1,2,3,4,5,6,7,8,9].

Over the past decade, the literature on cancer-derived exosomes has expanded rapidly. Tumor exosomes have been implicated in cell proliferation, epithelial-to-mesenchymal transition, extracellular matrix remodeling, angiogenesis, immune evasion, organotropic dissemination, and resistance to therapy. Their presence in accessible body fluids such as plasma, serum, urine, saliva, and ascites has made them particularly attractive for liquid biopsy applications. Unlike tissue biopsy, which samples a limited spatial portion of a heterogeneous lesion at a single time point, exosome-based analysis may in principle capture a more dynamic and system-wide portrait of tumor activity. This possibility is one of the main reasons that exosomal proteins and exosomal noncoding RNAs are now being explored as potential analytes for early detection, disease monitoring, prognostic stratification, and treatment-response assessment [1,3,4,5,6,7,8,9].

Despite this rapid growth, the literature remains highly fragmented. Many studies are organized around a single tumor type, a specific body fluid, a single cargo class, or a particular detection technology. This fragmentation has generated large inventories of candidate molecules but has made it difficult to determine which exosomal features are broadly conserved across cancers and which are specific to particular disease groups or tissue contexts. In addition, the exosome field is strongly affected by methodological heterogeneity. Isolation procedures, vesicle purity, body fluid source, analytical platform, and database selection can each alter the profile of reported proteins and RNAs. A review that merely lists molecules without considering these issues risks overstating specificity and underestimating technical variability [2,3,4,5,6,7,8,9,10].

This review addresses that gap through an explicitly comparative framework. The review examines exosomal proteins and microRNAs across eight cancer categories—blood, breast, colon, kidney, liver, lung, prostate, and stomach cancers—and further compares hematologic versus solid malignancies. The goal is not to claim definitive pathway activation from enrichment outputs alone, but to synthesize recurring biological themes, identify structured differences among disease contexts, and evaluate how integrated protein-and-microRNA profiling may contribute to cancer biology and translational oncology. Throughout, the discussion is embedded within each analytical section so that the figures are interpreted mechanistically, methodologically, and clinically rather than described in a purely catalog-like manner [4,5,6,7,8,9,10]. Building on this comparative framework, the present review is guided by an explicit central hypothesis: that cancer-derived exosomal cargo is organized into two separable layers—a conserved, lineage-independent core that reflects shared vesicle-biogenesis and trafficking machinery, and a variable, context-dependent layer that reflects tissue of origin and microenvironmental state. The specific mechanistic problem addressed here is therefore to determine which exosomal functions behave as cargo-selection invariants across cancers and which are tissue-driven variables. This hypothesis yields a falsifiable expectation: membrane-, vesicle-, and transport-associated functions should recur across all eight cancer categories, whereas tissue-restricted functional signatures should track disease group and body-fluid origin. The hematologic-versus-solid comparison is used as a direct test of this prediction.

## 2. Scope and Methodology of the Review

### 2.1. Scope and Rationale

This is a comparative review of the reported exosomal protein and microRNA (miRNA) cargo of eight cancer categories. To synthesize a fragmented literature on a common basis, the review is organized around a transparent, reproducible re-analysis of previously published exosomal molecules rather than around a narrative summary alone; this Section describes the scope of that synthesis and the procedures used to generate the comparative figures. The eight categories examined are: blood, breast, colon, kidney, liver, lung, prostate, and stomach cancer. Rather than performing new wet-laboratory experiments, previously reported exosomal molecules were retrieved from curated exosome repositories and re-analyzed within a single, uniform bioinformatic framework so that functional categories could be compared across tumor types on a common basis. Two molecular layers were examined in parallel: (i) exosomal proteins, interpreted through Gene Ontology (GO), Kyoto Encyclopedia of Genes and Genomes (KEGG) pathway mapping, and PANTHER protein class annotation; and (ii) exosomal miRNAs, interpreted through target-protein prediction followed by the same enrichment-based annotation. All analyses were descriptive and hypothesis-generating; no claim of causal pathway activation was inferred from enrichment output alone. This review is deliberately restricted to exosomes (small extracellular vesicles of endosomal origin, approximately 30–150 nm) and does not include microvesicles or large oncosomes. This restriction is both biological and data-driven: microvesicles bud directly from the plasma membrane and large oncosomes are shed by amoeboid tumor cells, so their biogenesis, size ranges, and cargo-sorting mechanisms differ substantially from those of exosomes, and pooling them would confound the cargo-selection question addressed here; in addition, the curated entries analyzed from ExoCarta and Vesiclepedia are predominantly annotated to exosomes/small extracellular vesicles, and the underlying studies rarely separate microvesicle- or oncosome-specific cargo. Because isolation methods cannot fully resolve these subpopulations, a residual degree of vesicle-type overlap remains an inherent limitation (Section 13).

### 2.2. Data Sources and Retrieval of Exosomal Cargo

Exosomal protein and miRNA entries were retrieved from established exosome-specific databases, principally ExoCarta and Vesiclepedia, which provide community-annotated compendia of molecules identified in exosomes and other extracellular vesicles. For each of the eight cancer categories, entries were filtered by the associated tissue or disease annotation and by species (Homo sapiens). Only molecules reported in studies describing exosomes or small extracellular vesicles were retained. Because repository content is updated continuously, the retrieval date is reported to allow future reconstruction of the dataset. Both ExoCarta https://www.exocarta.org/ and Vesiclepedia https://www.microvesicles.org/ were accessed on 5 December 2025. The eight cancer categories analyzed here were determined by data availability rather than by biological pre-selection: they are the tumor types for which ExoCarta and Vesiclepedia provided curated, human, exosome-annotated entries of sufficient size to support both protein and miRNA enrichment analysis under a single uniform pipeline. Tumor types with sparse entries, or with body-fluid sources too ambiguously annotated to permit consistent handling, were not included, because analyzing them on an unequal footing would reintroduce the methodological non-comparability this review is designed to avoid. This inclusion criterion is bounded by current repository coverage, and the resulting disease-selection dependence is discussed as a limitation in Section 13.

For each cancer category, the retrieved molecules were compiled into non-redundant protein and miRNA lists. Protein identifiers were harmonized to official UniProt/HGNC gene symbols arising from synonymous identifiers or from multiple contributing studies were collapsed so that each protein was counted once per cancer category. miRNA identifiers were standardized to current miRBase nomenclature; where legacy names were encountered, they were mapped to the corresponding current accession before de-duplication. The complete input lists for every cancer category are provided in the Supplementary Materials to permit independent verification and reproduction of all downstream analyses.

### 2.3. Counting Conventions and Dataset Composition

Reported counts refer to the number of unique molecules retained for each cancer category after harmonization and de-duplication, unless otherwise stated. The number of proteins analyzed per category was 523 (blood), 794 (breast), 113 (colon), 842 (kidney), 125 (liver), 191 (lung), 506 (prostate), and 549 (stomach), giving 523 proteins for hematologic malignancies and 3120 proteins for solid tumors in the grouped comparison. For miRNAs, two quantities are distinguished throughout this review: the number of unique exosomal miRNAs retrieved for each cancer category, and the number of miRNA–target interaction pairs generated when those miRNAs were mapped to predicted target proteins (Section 2.5). The number of unique miRNAs per category was 294 (blood), 14 (breast), 12 (colon), 5 (kidney), 11 (liver), 11 (lung), 34 (prostate), and 3 (stomach), corresponding to 350 unique miRNAs across all categories after de-duplication. The associated numbers of miRNA–target pairs were 472,016 (blood), 17,163 (breast), 6518 (colon), 6557 (kidney), 33,161 (liver), 24,164 (lung), 49,229 (prostate), and 18,417 (stomach). Because a small number of miRNAs can generate a large number of predicted interactions, these proportions are interpreted as reflecting predicted-interaction density rather than miRNA diversity. Complete per-category unique-miRNA and pair counts are provided in Table S1.

### 2.4. Functional Annotation of Exosomal Proteins

Non-redundant protein lists were functionally annotated using three complementary resources applied under identical settings for every cancer category. Gene Ontology terms for biological process, molecular function, and cellular component were assigned and summarized to describe the functional organization of each proteome. Protein class distribution was determined with the PANTHER classification system (Protein ANalysis THrough Evolutionary Relationships) via the PANTHER web server, using Homo sapiens as the reference organism. Pathway associations were obtained by KEGG pathway mapping using the KEGG Mapper tool, with gene symbols converted to KEGG identifiers prior to mapping. The KEGG Mapper tool is available at https://www.genome.jp/kegg/mapper.html and was accessed on 5 December 2025. For each annotation category, results are presented as the proportional distribution of proteins across terms or classes; where a protein mapped to more than one term, it was counted in each applicable category, and this multiplicity is reflected in the summarized distributions.

### 2.5. Target-Protein Prediction and Functional Annotation of Exosomal miRNAs

Because miRNAs act as post-transcriptional regulators rather than as direct structural or enzymatic effectors, their functional interpretation was based on predicted target proteins. For each cancer category, target genes of the retrieved miRNAs were predicted using TargetScan, miRDB, and experimentally supported interactions catalogued in miRTarBase. A predicted miRNA–target relationship was retained when supported by at least two of these three resources (i.e., predicted by TargetScan and miRDB and/or annotated as an experimentally validated interaction in miRTarBase); these miRNA–target pairs are the entries counted in Section 2.3. The resulting non-redundant target-protein lists were then annotated with the same GO and KEGG procedures described in Section 2.4, allowing the regulatory (miRNA) layer and the effector (protein) layer to be compared within one framework. The requirement for agreement of at least two of the three resources is grounded in their methodological complementarity: TargetScan is a sequence- and conservation-based predictor, miRDB applies a machine-learning model, and miRTarBase catalogues experimentally supported interactions. Requiring concordance across at least two of these orthogonal lines of evidence substantially reduces the algorithm-specific false positives that dominate any single predictor, which is why consensus (voting) approaches are widely used in miRNA-target analysis. We note, however, that a consensus rule is conservative by design and is therefore weighted toward specificity rather than sensitivity: a genuine interaction of a low-abundance but biologically critical miRNA that is documented in only one resource would not survive this filter. The reported miRNA–target counts should accordingly be read as high-confidence rather than exhaustive, and abundance-weighted or experimentally prioritized target selection is a natural extension for single-miRNA follow-up studies (see Section 13).

### 2.6. Comparative Analyses Across Cancer Categories

Two levels of comparison were performed using the annotated outputs described above. First, a pan-cancer comparison summarized proteins and miRNAs across all eight categories. Second, a disease-group comparison contrasted hematologic malignancies (blood cancer) with solid tumors (breast, colon, kidney, liver, lung, prostate, and stomach cancers) at the protein and miRNA levels. For every comparison, the identical GO, KEGG, and PANTHER pipeline was applied so that differences in functional distribution could be attributed to dataset composition rather than to differences in analytical method.

### 2.7. Statistical Interpretation and Methodological Limitations

Functional categories were summarized descriptively rather than through formal over-representation testing. Gene Ontology terms and protein classes were assigned with the PANTHER classification system via the PANTHER web server, and pathway associations were obtained by KEGG pathway mapping with the KEGG Mapper tool, using *Homo sapiens* as the reference organism. For each annotation category, results are expressed as the count or proportional distribution of proteins (or predicted miRNA targets) across terms and classes, and the figures display the most frequently represented terms in each category. Because no formal enrichment statistics or multiple-testing-adjusted *p*-values were computed, these distributions indicate structured association within the dataset rather than direct evidence of pathway activation in vivo, and this interpretive limit is retained throughout the review. Three additional constraints follow directly from the data and methods. (i) Repository content reflects prior research intensity, so larger inventories for some cancer types (for example, kidney and breast) partly reflect study availability rather than intrinsic biological richness. (ii) miRNA target assignment is prediction-dependent and sensitive to database choice and threshold, so miRNA-derived functional patterns are hypothesis-generating rather than confirmatory. (iii) Because retrieval aggregates studies that differ in isolation method, vesicle purity, body-fluid source, and analytical platform, cross-category comparisons are only as robust as the comparability of the underlying primary studies. These limitations are examined further in Section 13.

### 2.8. Data Availability

All input protein and miRNA lists, the harmonized datasets, and the full GO/KEGG/PANTHER output tables underlying Tables S2–S5 are provided as Supplementary Materials. No new primary (wet-laboratory) data were generated; all analyzed molecules were obtained from the publicly accessible repositories cited in Section 2.2. 

## 3. Biological Basis of Cancer-Derived Exosomes

**Figure 1:**
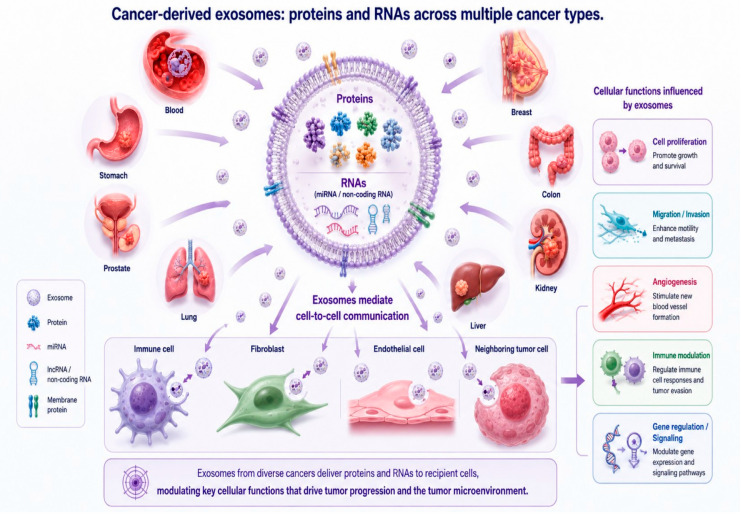
Schematic overview of cancer-derived exosomes as intercellular carriers of proteins and RNAs across multiple cancer types.

A biologically meaningful interpretation of exosomal cargo begins with exosome biogenesis. Exosomes arise from endosomal maturation, inward budding of the endosomal membrane, and the formation of intraluminal vesicles within multivesicular bodies, followed by fusion of these bodies with the plasma membrane. This sequence allows cargo selection to occur at multiple points, involving ESCRT-dependent and ESCRT-independent mechanisms, tetraspanin-enriched microdomains, membrane lipid composition, and trafficking regulators such as Rab family proteins. In cancer, these pathways are frequently altered by oncogenic signaling, hypoxia, oxidative stress, metabolic rewiring, and therapy-induced adaptation. Therefore, the molecular contents of tumor-derived exosomes should not be viewed as passive leftovers from the cytoplasm, but as a selectively assembled molecular output shaped by tumor-state-dependent trafficking decisions [4,5,6,7,8,9,10,11,12,13]. The implications of selective packaging are profound. If cargo composition is regulated rather than random, then exosomes can provide biologically interpretable information rather than merely reflecting cellular debris. Tumor-derived vesicles may therefore function both as records of tumor state and as effectors that reshape recipient-cell behavior. This duality is one of the core reasons that exosomes have attracted sustained interest in oncology: they are simultaneously mechanistic participants in disease progression and candidate analytes for minimally invasive clinical interrogation [1,2,3,4,5,6,7,8,9]. As summarized schematically in Figure 1, cancer-derived exosomes can mediate communication among tumor cells, immune cells, fibroblasts, endothelial cells, and other components of the tumor microenvironment. Following receptor engagement, membrane fusion, or endocytic uptake, exosomes may alter transcriptional programs, signaling cascades, and metabolic states in recipient cells. In this way, vesicle transfer can contribute to immune modulation, stromal activation, angiogenesis, extracellular matrix remodeling, and the phenotypic plasticity of adjacent malignant cells [1,2,3,4,5,6,7,8,9]. At the same time, the biological meaning of exosomal cargo cannot be separated from technical reality. Vesicles in clinical and experimental preparations are influenced by body fluid source, enrichment strategy, sample handling, and reporting quality. Therefore, exosomal signatures should be interpreted within the broader ecology of tumor–microenvironment communication and extracellular vesicle standardization rather than as isolated molecule lists [2,4,5,7,8,9,10]. Importantly, cargo loading is not a single uniform process but proceeds through several partly independent routes—ESCRT-dependent sorting, tetraspanin- and ceramide/nSMase2-associated pathways, and RNA binding protein guided sorting of miRNAs—whose relative contributions can differ with cell lineage, oncogenic signaling, and microenvironmental stress. This mechanistic heterogeneity provides a biological rationale for the disease-group differences observed later in this review: if hematologic and solid tumors preferentially engage different loading machinery, their exosomal cargo profiles should differ in structured ways rather than randomly. We treat this as a mechanistic interpretation to be tested rather than as an established cause.

Exosomes released from eight cancer types, including blood, breast, colon, kidney, liver, lung, prostate, and stomach cancers, are depicted as membrane-enclosed vesicles carrying proteins and RNA species, including miRNAs and other non-coding RNAs. These vesicular cargos may reflect the molecular state of their parental cancer cells and can be transferred to recipient cells such as immune cells, fibroblasts, endothelial cells, and neighboring tumor cells. Through this intercellular transfer, cancer-derived exosomes may modulate key cellular functions, including proliferation, migration, invasion, angiogenesis, immune regulation, and gene expression, thereby contributing to tumor progression and remodeling of the tumor microenvironment.

## 4. Rationale for Integrating Protein and microRNA Layers

Beyond the procedural steps described in Section 2, the interpretive logic of this review rests on integrating two molecular layers—proteins and microRNAs—within a common functional framework. Exosomal proteins are interpreted using Gene Ontology biological process, molecular function, and cellular component annotation together with KEGG pathway mapping and PANTHER protein class classification. This strategy allows exosomal proteomes to be read not simply as inventories of molecules, but as organized functional systems that can be compared across cancer contexts [10].

MicroRNAs are incorporated through a parallel but not identical strategy. Because miRNAs act primarily as post-transcriptional regulators rather than direct structural or enzymatic effectors, their interpretation requires target-protein prioritization followed by enrichment-based annotation. Integrating these analyses makes it possible to compare immediate functional effectors and regulatory signaling layers within the same review framework, while also acknowledging the prediction-sensitive nature of miRNA target inference [3,4,5,6,7,8,9,10]. 

## 5. Pan-Cancer Exosomal Proteome Across Eight Cancer Types

**Figure 2:**
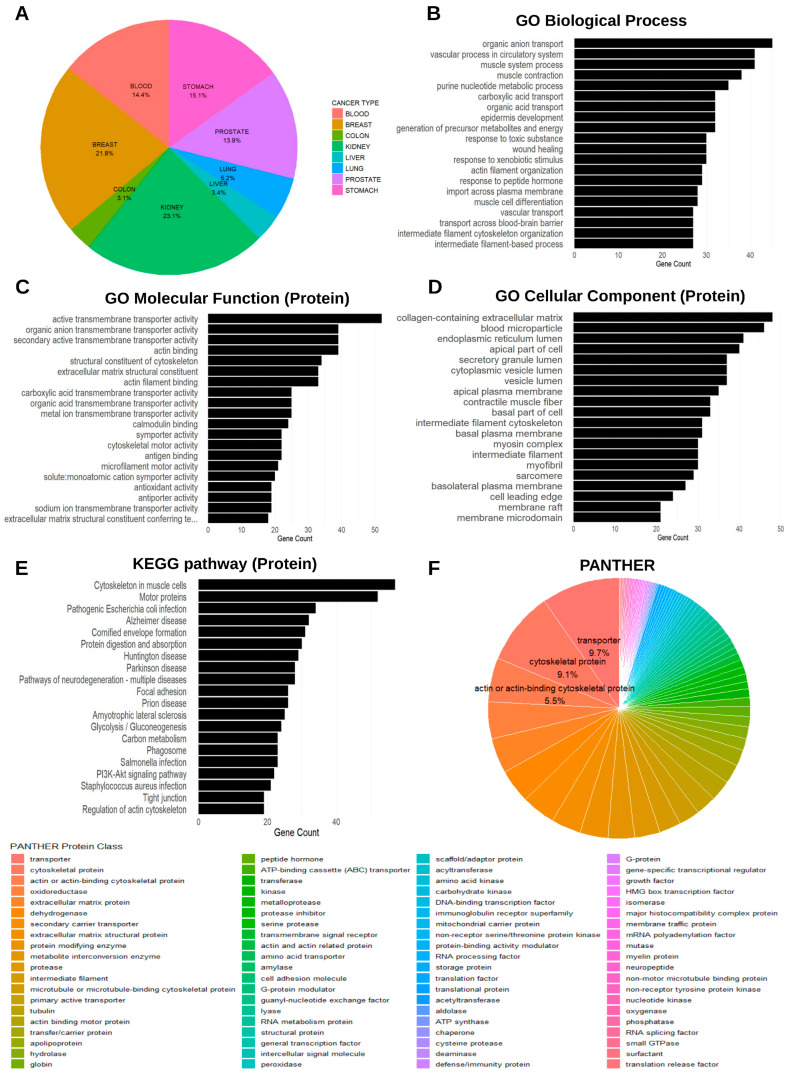
Protein-based bioinformatic analysis of exosomes derived from diverse cancer types. (**A**) Pie chart showing the proportional distribution of exosomal proteins identified across eight cancer types: blood cancer, breast cancer, colon cancer, kidney cancer, liver cancer, lung cancer, prostate cancer, and stomach cancer. The number of proteins included for each cancer type was as follows: blood cancer, 523; breast cancer, 794; colon cancer, 113; kidney cancer, 842; liver cancer, 125; lung cancer, 191; prostate cancer, 506; and stomach cancer, 549. (**B**) Functional classification of exosomal proteins based on Gene Ontology biological process terms. (**C**) Functional classification of exosomal proteins based on Gene Ontology molecular function terms. (**D**) Functional classification of exosomal proteins based on Gene Ontology cellular component terms. (**E**) Protein class distribution of exosomal proteins analyzed using PANTHER (Protein Analysis Through Evolutionary Relationships). (**F**) KEGG pathway classification of exosomal proteins, highlighting molecular-level biological pathways associated with intracellular metabolism, gene regulation, protein interactions, and related cellular processes.

Figure 2 presents the first major analytical layer: a pan-cancer overview of exosomal proteins identified across eight malignancy categories. The summarized dataset includes 523 proteins from blood cancers, 794 from breast cancer, 113 from colon cancer, 842 from kidney cancer, 125 from liver cancer, 191 from lung cancer, 506 from prostate cancer, and 549 from stomach cancer. These values immediately communicate an important principle: exosomal proteome datasets are not uniform in scale. Some categories, such as kidney and breast cancer, are represented by substantially larger proteomic inventories than colon or liver cancer. This difference should not be interpreted narrowly as a biological conclusion; it likely reflects a composite effect of dataset availability, sampling depth, biological variability, and the uneven intensity of prior exosome research across tumor types. Nevertheless, these numerical asymmetries matter because they influence which functional categories become detectable in cross-cancer comparisons [1,3,4,5,6,7,8,9,10]. A scientifically useful interpretation of this figure begins by distinguishing abundance from organization. The central value of Figure 2 is not merely that it counts proteins, but that it places them into an explicitly functional architecture. Pan-cancer exosome analysis is most informative when it asks whether proteins converge on reproducible biological states rather than whether a single molecule uniquely defines a tumor. This perspective is particularly important for biomarker development, because clinical assays often perform better when they capture coordinated molecular programs rather than relying on one putatively specific analyte [1,4,5,6,7,8,9,10]. 

The biological process layer is especially informative because it organizes the pan-cancer protein landscape around coordinated cellular activities. Across diverse cancers, exosomal proteins tend to cluster around processes related to signaling, transport, regulation of cellular responses, cytoskeletal organization, and extracellular interaction. These recurrent themes are biologically plausible because tumor-derived vesicles must participate in communication with stromal cells, survival under stress, and adaptation to changing microenvironmental conditions [1,3,4,5,6,7,8,9,10]. 

The molecular function and cellular component layers complement this interpretation by adding biochemical and structural context. Functional classes associated with binding, catalytic activity, transporter-related roles, membrane-linked compartments, vesicle-associated structures, and extracellular interfaces are all relevant to the biology of cancer exosomes. Such patterns reinforce the notion that exosomal proteins are assembled into coherent biological modules rather than representing a random spillover of intracellular contents [10].

KEGG pathway mapping and PANTHER classification add another layer of value by framing the exosomal proteome in terms of signaling logic and protein family structure. These approaches are particularly useful in a review setting because they help distinguish recurring, cross-cancer organizational themes from isolated protein observations. At the same time, pathway enrichment should be interpreted cautiously: it suggests structured association, not definitive activation. Thus, Figure 2 is best viewed as a biologically organized atlas of pan-cancer exosomal proteins rather than as direct proof of deterministic pathway behavior [1,6,7,8,9,10].

## 6. Cancer-Type-Specific Considerations in Exosomal Protein Studies

Hematologic malignancies occupy a special place in exosome biology because malignant cells develop within a fluid, immune-rich environment rather than within a fixed epithelial architecture. Exosomal proteins from leukemias, lymphomas, and related disorders are therefore particularly relevant to immune-cell crosstalk, marrow niche conditioning, cytokine responsiveness, and therapy resistance. Compared with solid tumors, blood cancer exosomes may reflect lineage-associated signaling and circulation-linked communication more strongly than matrix-centric remodeling. This difference is one reason why hematologic malignancy exosomes should not be analyzed solely through a solid-tumor conceptual lens [11,12,13].

Breast cancer exosome studies often emphasize heterogeneity at multiple levels, including hormone receptor status, HER2 signaling, metastatic preference, and treatment response. Exosomal proteins in this setting are frequently discussed as potential biomarkers of subtype-specific biology and as mediators of intercellular crosstalk within a complex stromal and immune microenvironment. The large breast cancer protein inventory summarized in the present review is therefore consistent with a literature that has long regarded breast cancer exosomes as both mechanistically important and clinically promising [14,15,16,17,18,19].

Colorectal cancer provides a distinct exosomal context dominated by epithelial polarity, barrier interfaces, inflammatory signaling, microbial exposure, and early metastatic interaction with the liver. Exosomal proteins in colorectal cancer have been linked to invasion, stromal remodeling, progression, and biomarker development. These features make colorectal cancer an important comparator when considering how organ-specific epithelial biology shapes the content and interpretation of extracellular vesicles [20,21,22,23,24,25,26].

Kidney cancer contributes one of the larger protein inventories in the present review, which is noteworthy given the importance of renal physiology, vascular signaling, and urinary biomarker access in this disease context. Exosomal proteins in renal cell carcinoma have been examined both for their mechanistic relevance to tumor progression and for their potential diagnostic value, particularly because kidney-derived vesicular material may be detectable in urine as well as blood [27,28,29,30,31]. It should be emphasized, however, that current repositories do not annotate the source biofluid uniformly for every entry, so the kidney protein set analyzed here likely combines urinary and systemic (plasma/serum) exosomes; the features discussed are therefore attributed to exosomes reported in this cancer rather than to a specific biofluid compartment (see Section 13).

Liver cancer exosomal proteins are commonly interpreted against a background of chronic inflammation, cirrhosis, metabolic dysfunction, viral hepatitis, and a highly vascular organ microenvironment. In hepatocellular carcinoma, exosomal cargo has been associated with progression, recurrence, metastasis, and drug resistance, while also being explored as a candidate biomarker source. These features underscore how strongly disease context can shape the biological meaning of exosomal protein data [32,33,34].

Lung cancer exosome biology is shaped by airway exposure, inflammatory signaling, stromal diversity, and early metastatic competence. Exosomal proteins in lung cancer have attracted interest because minimally invasive biomarkers would be especially valuable in a disease often diagnosed late and characterized by rapid clinical evolution. The literature therefore frequently interprets lung cancer exosomes in both mechanistic and translational terms [35,36,37,38,39].

Prostate cancer exosomal proteomics has special translational relevance because of the long-standing interest in urinary biomarkers for urologic malignancies. Exosomal proteins and noncoding RNAs from prostate cancer have been investigated in both plasma and urine, with the goal of improving detection, aggressiveness assessment, and disease monitoring. This body-fluid accessibility gives prostate cancer a particularly favorable setting for exosome-based liquid biopsy development [40,41,42,43,44,45,46,47,48]. As for kidney cancer, the prostate exosome entries aggregated from public repositories are not uniformly resolved by biofluid, and are likely to include both urinary and blood-derived vesicles; we therefore do not claim that the prostate protein features are exclusively urinary in origin.

Stomach cancer exosomes are frequently considered in relation to inflammation, epithelial barrier disruption, stromal reprogramming, and the progression from chronic mucosal injury to malignant transformation. Gastric cancer-associated exosomal proteins and RNAs have been studied as mediators of progression and as candidate biomarkers for early detection and disease monitoring, highlighting the convergence of mechanistic and diagnostic interest in this cancer type [49,50,51].

## 7. Comparative Exosomal Proteome in Hematologic and Solid Malignancies

**Figure 3:**
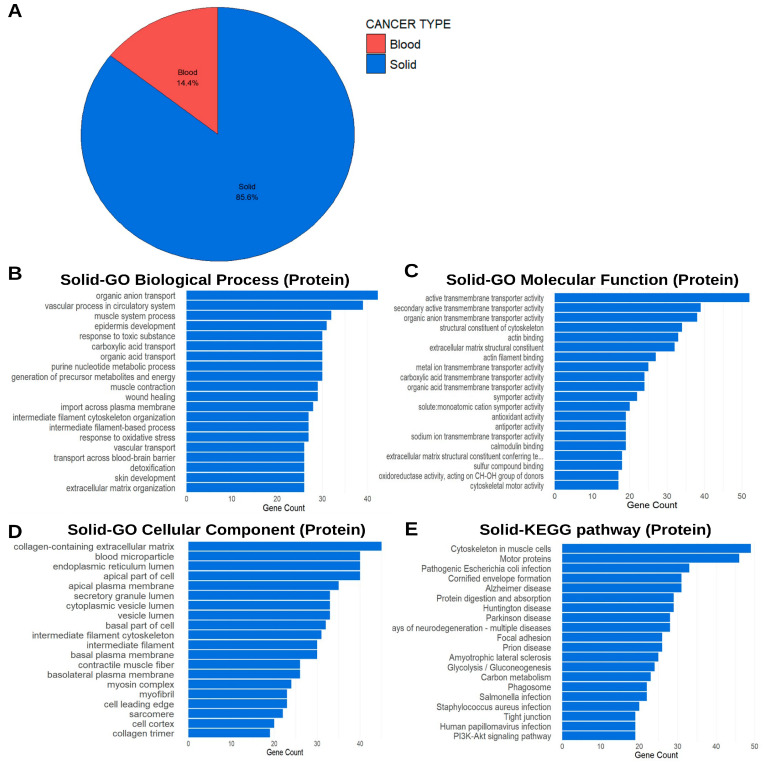

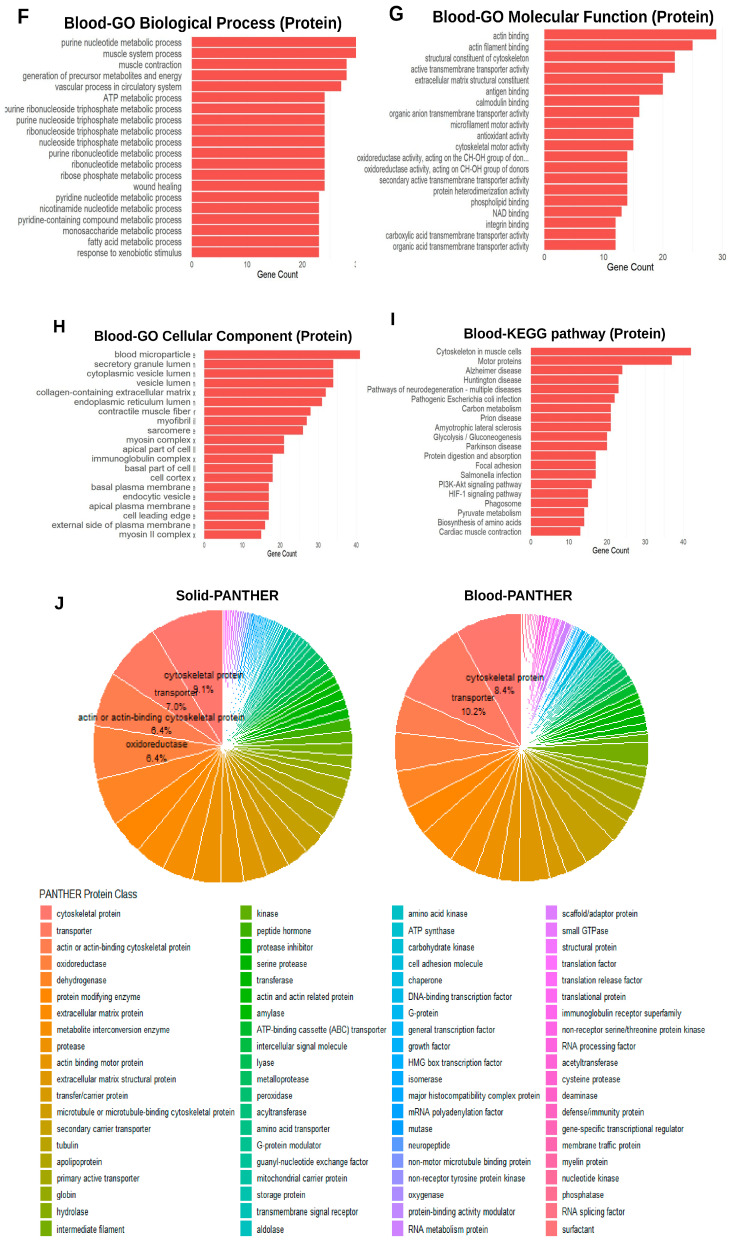
Bioinformatic analysis of exosomal proteins identified in hematologic malignancies and solid tumors. (**A**) Pie chart showing the proportional distribution of exosomal proteins identified in hematologic malignancies and solid tumors. A total of 523 proteins were included from hematologic malignancies, whereas 3120 proteins were included from solid tumors. (**B**–**E**) Bioinformatic classification of proteins identified in exosomes derived from solid tumors, including Gene Ontology biological process analysis (**B**), Gene Ontology molecular function analysis (**C**), Gene Ontology cellular component analysis (**D**), and KEGG pathway classification (**E**). (**F**–**I**) Bioinformatic classification of proteins identified in exosomes derived from hematologic malignancies, including Gene Ontology biological process analysis (**F**), Gene Ontology molecular function analysis (**G**), Gene Ontology cellular component analysis (**H**), and KEGG pathway classification (**I**). (**J**) PANTHER (Protein Analysis Through Evolutionary Relationships) analysis of exosomal proteins identified in solid tumors (**B**–**E**) and hematologic malignancies (**F**–**I**), showing protein classification based on evolutionary relationships.

Figure 3 compares hematologic malignancies and solid tumors at the proteome level, introducing a disease-ecology dimension to exosome analysis. In the current dataset, exosomal proteins from blood cancers total 523, whereas those assigned to solid tumors total 3120. This difference is substantial and immediately suggests that solid-tumor exosome studies collectively sample a broader protein repertoire, whether because of deeper literature accumulation, more diverse tissue sources, or intrinsically richer heterogeneity in tumor–stroma interactions. Yet the real scientific importance of this comparison lies less in the raw counts than in what the contrast reveals about disease context. Hematologic malignancies operate within circulating, marrow-based, or lymphoid ecosystems, whereas solid tumors are embedded within tissue architecture shaped by extracellular matrix, fibroblasts, endothelial cells, perivascular compartments, and organ-specific niches [1,6,7,8,9,10,11,12,13].

These differences in ecological setting are likely to shape exosomal protein content in ways that go beyond the identity of the malignant cell itself. Blood cancer exosomes may preferentially encode lineage-associated signaling, immune modulation, and circulation-linked communication, whereas solid-tumor vesicles may more strongly reflect tissue remodeling, epithelial–stromal interaction, matrix engagement, and organ-specific metastatic adaptation. The value of the comparison therefore lies in revealing how disease context conditions the organization of exosomal functions [1,6,7,8,9,11,12,13].

Methodologically, this section also highlights why a shared analytical framework is essential. If blood cancer and solid-tumor datasets were compared only by raw lists of proteins, the interpretation would quickly collapse into descriptive cataloging. By using common GO, KEGG, and PANTHER approaches, Figure 3 provides a structured basis for determining whether disease-group-specific biology is encoded not only in individual molecules, but also in the higher-level architecture of exosomal functions and protein classes [10].

From a clinical perspective, the blood-versus-solid comparison argues against a universal exosome biomarker template. A panel that performs well in a hematologic malignancy may not generalize directly to a solid tumor, because the biological demands and tissue ecologies of these settings differ substantially. Future validation efforts should therefore aim to identify combinations of vesicle-associated proteins that maximize discrimination while remaining robust across sampling workflows and disease contexts [4,5,6,7,8,9,11,12,13].

## 8. Pan-Cancer Exosomal microRNA Landscape Across Eight Cancer Types

**Figure 4:**
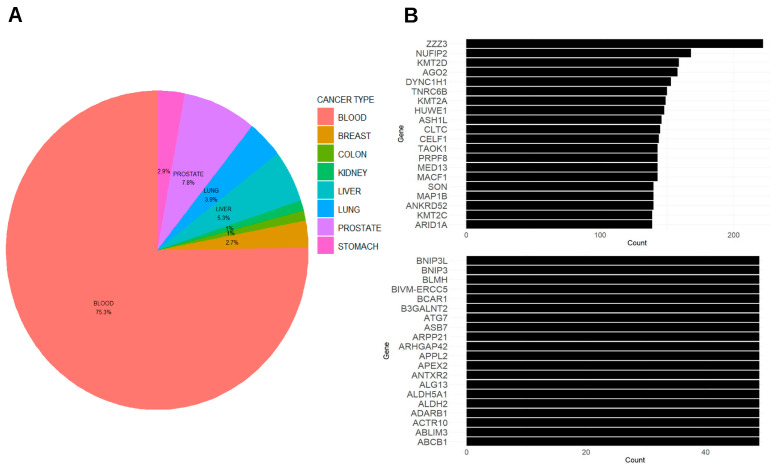

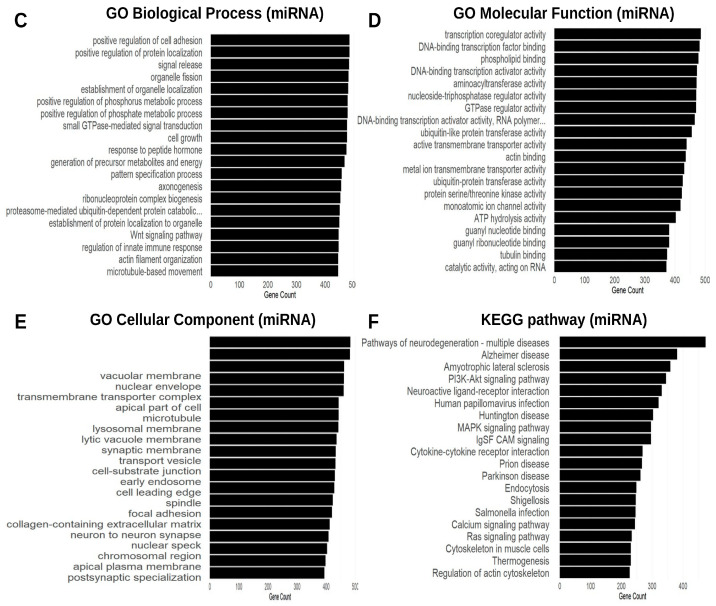
miRNA-based bioinformatic analysis of exosomes derived from diverse cancer types. (**A**) Pie chart showing the proportional distribution of exosomal miRNAs identified across eight cancer types: blood cancer, breast cancer, colon cancer, kidney cancer, liver cancer, lung cancer, prostate cancer, and stomach cancer. The chart summarizes the distribution of miRNA–target interaction pairs, not of unique miRNAs. The number of pairs for each cancer type was as follows: blood cancer, 472,016; breast cancer, 17,163; colon cancer, 6518; kidney cancer, 6557; liver cancer, 33,161; lung cancer, 24,164; prostate cancer, 49,229; and stomach cancer, 18,417. These pairs were derived from 294, 14, 12, 5, 11, 11, 34, and 3 unique exosomal miRNAs, respectively (350 unique miRNAs across all categories; Table S1). (**B**) Analysis of miRNA-associated target proteins, showing the top 20 most frequently identified proteins and the bottom 20 least frequently identified proteins. (**C**–**F**) Bioinformatic classification of miRNAs identified across the eight cancer types, including Gene Ontology biological process analysis (**C**), Gene Ontology molecular function analysis (**D**), Gene Ontology cellular component analysis (**E**), and KEGG pathway classification (**F**).

Figure 4 extends the review from the layer of immediate molecular effectors to the layer of regulatory signaling by summarizing exosomal microRNAs across the same eight cancer types. In the eight-cancer comparison, the eight cancer categories are represented by 350 unique exosomal miRNAs in total, ranging from as few as 3–14 unique miRNAs in several solid tumors to 294 in blood cancer. When these miRNAs are mapped to predicted targets, they generate markedly larger and highly uneven numbers of miRNA–target pairs (472,016 for blood cancer, and 6518–49,229 across the solid-tumor categories; Table S1). The very large blood-cancer pair count therefore reflects the number of predicted interactions rather than an unusually large miRNA repertoire, a distinction that is essential to avoid misreading Figure 4A as a map of miRNA diversity. The scale and asymmetry of these values differ markedly from the protein datasets and are themselves informative. The very large blood-cancer pair count most plausibly reflects deeper aggregation and denser reporting of predicted interactions in hematologic settings rather than a fundamentally larger set of distinct miRNAs. Whatever the cause, this disparity reminds the reader that miRNA datasets are especially vulnerable to differences in platform, database coverage, normalization strategy, and study aggregation [3,4,5,6,7,8,9,10]. To make these miRNAs directly usable for biomarker discovery, the identity (miRBase ID) of every exosomal miRNA included for each cancer type is now provided in Supplementary Table S6, complementing the target-protein view in Figure 4B. Comparison of these identities across cancers is itself informative. No single miRNA is shared by all eight categories in the present dataset, and the maximum overlap observed is three cancer types; 29 miRNAs are shared by two or more cancers, including several with well-documented oncogenic roles (for example, hsa-let-7b/c-5p, hsa-miR-125a/b-5p, hsa-miR-30a-5p, hsa-miR-375, and hsa-miR-28-5p), whereas the large majority are cancer-restricted (for instance, 276 miRNAs unique to blood cancer and 20 to prostate cancer). The absence of a universal, all-cancer onco-miR in these curated exosomal datasets is a meaningful observation in its own right: recurrent exosomal miRNA signals appear to be shared at the level of small, overlapping subsets and canonical onco-miRs rather than as a single pan-cancer miRNA, a pattern that also reflects the uneven depth of miRNA reporting across tumor types.

Even with that caveat, the biological importance of exosomal miRNAs is substantial. Unlike proteins, which often act as direct structural or enzymatic effectors, miRNAs operate through post-transcriptional regulation and can reprogram recipient cells by shifting transcript stability and downstream protein abundance. This gives exosomal miRNAs a distinctive role in tumor communication, allowing vesicles to transmit not only a record of current tumor state but also regulatory instructions that may shape future cellular behavior [2,3,4,5,6,7,8,9].

The recurrent association of miRNA target networks with signaling, transcription-related processes, vesicle-associated structures, and disease-relevant pathways makes biological sense in the context of cancer progression. Vesicle-transferred miRNAs have been linked to immune escape, stromal crosstalk, metastatic niche formation, angiogenesis, and treatment adaptation. Thus, target-prioritized enrichment analysis provides a useful bridge between raw miRNA abundance and plausible biological consequence, even though it remains prediction-dependent [3,4,5,6,7,8,9,10]. 

At the same time, miRNA interpretation requires special caution. Prediction-based target assignment is sensitive to the database used, the stringency of selection criteria, and the biological context in which targets are evaluated. For this reason, miRNA-associated enrichment should be treated as a hypothesis-generating interpretive layer rather than as direct proof of exosome-mediated regulation. The value of Figure 4 lies in its capacity to structure questions for future validation rather than to settle those questions definitively [3,4,5,6,7,8,9,10]. Incorporating miRNAs alongside proteins prevents exosome biology from being equated with proteomics alone. Cancer-derived vesicles are composite communication units, and capturing both effectors and regulatory cargo layers within a unified framework provides a more complete portrait of tumor communication [3,4,5,6,7,8,9,10].

## 9. Cancer-Type-Specific Considerations in Exosomal microRNA Studies

In hematologic malignancies, exosomal microRNAs are often discussed in the context of bone marrow niche conditioning, immune evasion, leukemia–stroma interaction, and acquisition of treatment resistance. Because malignant hematopoietic cells are already embedded within a mobile and immune-competent compartment, their exosomal miRNAs may be especially important for fine-tuning the surrounding cellular ecosystem. This feature makes blood cancer exosomal miRNAs attractive not only as biomarkers but also as candidate mediators of disease persistence and relapse [11,12,13].

Breast cancer exosomal microRNAs have been studied extensively because of their association with subtype heterogeneity, endocrine resistance, metastatic progression, and circulating biomarker potential. The literature frequently treats vesicle-associated miRNAs as both disease classifiers and mechanistic mediators of microenvironmental communication, thereby making breast cancer one of the best-developed settings for translational exosome RNA research [15,16,17,18,19].

Colorectal cancer exosomal microRNAs are often interpreted in relation to invasion, epithelial plasticity, inflammatory signaling, liver-directed dissemination, and non-invasive biomarker development. In this context, vesicle-associated miRNAs provide a conceptual link between local tumor biology and systemic communication, especially when explored in relation to gastrointestinal tumor progression and metastasis [20,21,22,23,24,25,26].

Kidney cancer exosomal microRNAs are especially interesting because urine offers a non-invasive sampling route that is directly relevant to disease detection and monitoring. In renal cell carcinoma, vesicle-associated RNAs may capture both tumor biology and organ-proximal biomarker accessibility, making this cancer type an important example of how clinical specimen context can shape exosome research priorities [27,28,30,31].

Liver cancer exosomal microRNAs are shaped by a disease context characterized by chronic inflammation, fibrosis, viral hepatitis, cirrhosis, and strong vascular coupling. Their interpretation therefore often bridges tumor biology with background liver pathology. This complexity also explains why hepatocellular carcinoma exosomal RNAs are discussed simultaneously as mechanistic mediators and as candidate biomarkers for prognosis, recurrence, and treatment response [32,33,34].

Lung cancer exosomal microRNAs are frequently studied because lung cancer diagnosis and monitoring would benefit strongly from minimally invasive biomarkers. Vesicle-associated miRNAs in this setting have been linked to tumor progression, clinical stratification, and actionable disease monitoring, particularly in the context of rapidly evolving systemic disease [35,36,37,38,39].

Prostate cancer exosomal microRNAs have drawn interest both in plasma and in urine because of their potential to support non-invasive diagnosis, prognosis, aggressiveness assessment, and monitoring. The large body of urologic exosome research makes prostate cancer one of the most clinically mature examples of liquid biopsy-oriented extracellular vesicle investigation [40,41,42,43,44,45,46,47,48].

Stomach cancer exosomal microRNAs may capture a biology shaped by chronic mucosal inflammation, epithelial transformation, and stromal reprogramming. They have been explored as participants in disease progression and as diagnostic or prognostic biomarkers, illustrating once again how mechanistic and translational interests converge in cancer exosome research [49,50,51].

## 10. Comparative Exosomal microRNAs in Hematologic and Solid Malignancies

**Figure 5:**
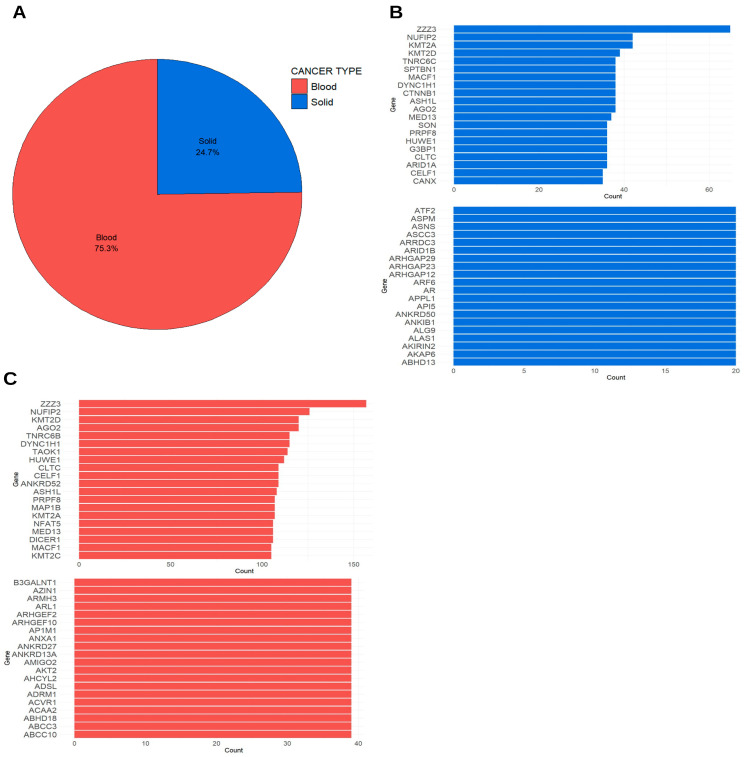

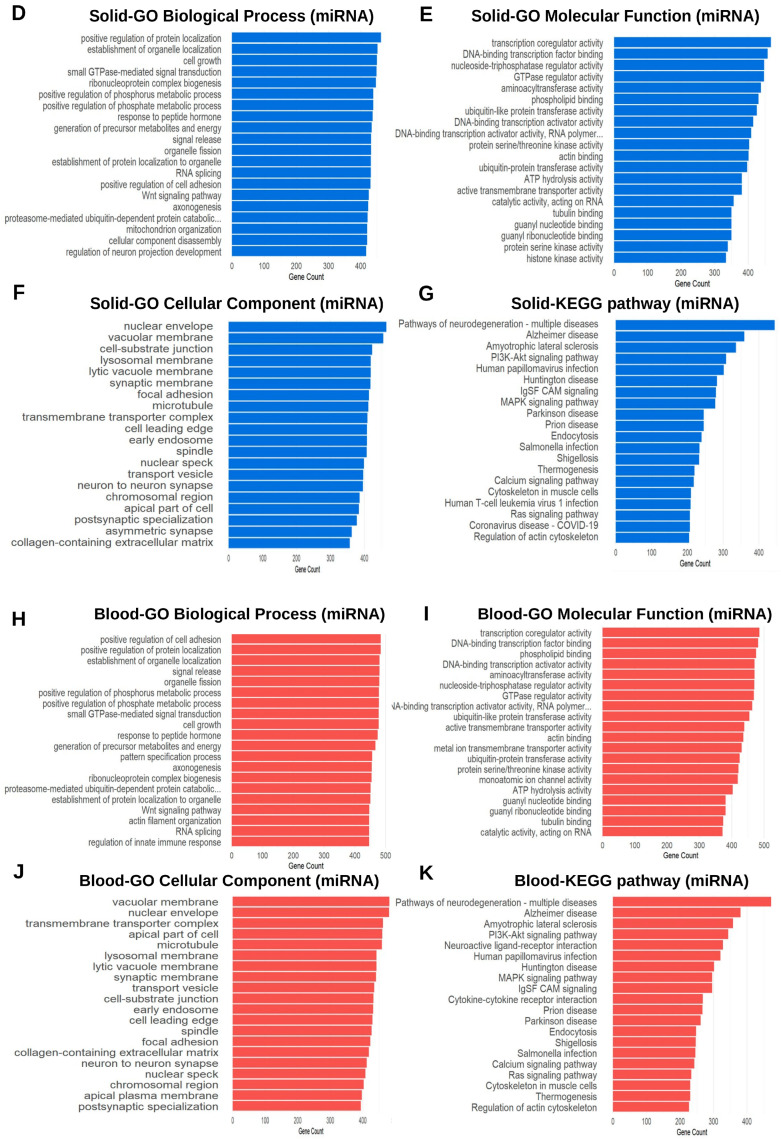
Bioinformatic analysis of exosomal miRNAs identified in hematologic malignancies and solid tumors. (**A**) Pie chart showing the proportional distribution of exosomal miRNAs identified in hematologic malignancies and solid tumors. The chart summarizes miRNA–target interaction pairs. A total of 472,016 pairs (from 294 unique miRNAs) were included from hematologic malignancies, whereas 155,209 pairs (from 74 unique miRNAs) were included from solid tumors (Table S1). (**B**) Analysis of miRNA-associated target proteins identified in solid tumors, showing the top 20 most frequently identified proteins and the bottom 20 least frequently identified proteins. (**C**) Analysis of miRNA-associated target proteins identified in hematologic malignancies, showing the top 20 most frequently identified proteins and the bottom 20 least frequently identified proteins. (**D**–**G**) Bioinformatic classification of miRNAs identified in solid tumors, including Gene Ontology biological process analysis (**D**), Gene Ontology molecular function analysis (**E**), Gene Ontology cellular component analysis (**F**), and KEGG pathway classification (**G**). (**H**–**K**) Bioinformatic classification of miRNAs identified in hematologic malignancies, including Gene Ontology biological process analysis (**H**), Gene Ontology molecular function analysis (**I**), Gene Ontology cellular component analysis (**J**), and KEGG pathway classification (**K**).

Figure 5 completes the comparative framework by extending the blood-versus-solid analysis to exosomal microRNAs. This comparison becomes especially informative because it asks whether disease-group-specific biology is evident not only in the executors of function, but also in the regulators of future cell states. The two disease groups differ sharply in predicted-interaction density—a difference driven partly by uneven reporting, as noted above—yet the comparative logic remains biologically valuable. Hematologic malignancies and solid tumors are expected to differ not only in exosomal proteins, but also in how vesicle-associated regulatory RNAs are deployed within their respective microenvironments. Blood cancer exosomes may preferentially support immune-cell communication, marrow adaptation, and persistence-related signaling, whereas solid-tumor exosomes may more strongly encode matrix interaction, stromal reprogramming, angiogenesis, and organotropic dissemination [3,4,5,6,7,8,9,11,12,13].

The use of target-protein prioritization followed by GO and KEGG analysis provides a practical way to compare these regulatory architectures. Although this strategy does not eliminate the uncertainties of target prediction, it enables a more structured cross-disease interpretation than would be possible from raw miRNA lists alone. This structured comparison is more informative than exhaustive molecule cataloging because it reveals interpretable regulatory tendencies at the systems level [3,4,5,6,7,8,9,10]. Translationally, future exosome assays should probably be multi-omic. A blood-versus-solid classifier built only on proteins may miss disease-group-specific regulatory patterns captured by miRNAs, while a miRNA-only assay may overlook the immediate functional phenotype encoded by proteins. The strongest biomarker strategies are therefore likely to emerge from integrated vesicle profiling rather than from single-cargo approaches [3,4,5,6,7,8,9].

## 11. Integrating Protein and microRNA Cargo into a Unified Biological Model

One of the most important conceptual messages of this review is that exosomal proteins and exosomal miRNAs should not be interpreted as independent parallel lists. They occupy different positions within a single communication system. Proteins often represent the machinery of vesicle structure, extracellular interaction, receptor engagement, enzymatic activity, or immediate signaling capacity. MicroRNAs, by contrast, encode a slower but potentially broader regulatory influence by shifting transcript and protein expression patterns in recipient cells. When both layers are analyzed together, exosomes become visible as composite biological entities containing both effectors and instructions. This integrated view is more faithful to tumor biology than a single-analyte perspective, particularly when synthesizing evidence across multiple cancer types [3,4,5,6,7,8,9,10]. Crucially, applying one identical enrichment pipeline to all eight cancers makes visible two cross-cancer regularities that are not apparent from single-dataset annotation: first, a recurrent, lineage-independent functional core—dominated by membrane, vesicle, and transport-associated categories—that is conserved across every cancer examined and is consistent with shared constraints on vesicle-mediated cargo selection; and second, a set of disease-group-specific signatures, most clearly resolved in the hematologic-versus-solid contrast, indicating that tissue context reshapes the variable layer of exosomal cargo. These two observations—a conserved core overlaid by context-dependent variation—are the principal incremental contribution of the unified re-analysis, and we present them as hypothesis-generating structure rather than as proof of pathway activation.

The interaction between these layers also has consequences for interpretation. A protein enrichment pattern that suggests extracellular interaction, membrane localization, or transporter activity does not simply coexist with an miRNA enrichment pattern involving transcription-related or signaling-associated targets. Instead, these layers may represent different temporal aspects of a coordinated vesicle strategy: proteins may shape uptake, interaction, and immediate signaling, while miRNAs may shape longer-term reprogramming of recipient cells. Such a framework is biologically plausible and helps explain why integrated analysis may outperform single-layer interpretation [2,3,4,5,6,7,8,9,10].

This integrated perspective also improves translational logic. Biomarker development often suffers when analyte classes are evaluated in isolation. A protein panel may provide structural and pathway-proximal information, whereas a miRNA panel may reflect regulatory plasticity, stress response, or metastatic adaptation. Combining these layers is therefore more likely to capture the multidimensional nature of tumor behavior than relying on one cargo class alone [4,5,6,7,8,9].

Importantly, a unified model does not require the field to claim that every exosome carries a stable and perfectly integrated molecular program. Heterogeneity remains intrinsic to extracellular vesicle biology. However, jointly interpreting exosomal proteins and miRNAs is better aligned with biological complexity and therefore more likely to generate clinically relevant hypotheses for future validation [2,3,4,5,6,7,8,9,10].

## 12. From Comparative Profiling to Clinical Implementation

The translational significance of cancer-derived exosomes arises from the convergence of three properties: accessibility, molecular richness, and biological relevance. First, exosomes circulate in clinically obtainable body fluids, enabling repeated and minimally invasive sampling. Second, their cargo includes multiple classes of analytes, including proteins, miRNAs, and other RNAs, allowing multi-layered characterization of tumor state. Third, because vesicles are released by living cells and can be shaped by stress, therapy, hypoxia, immune interaction, and metastatic adaptation, their contents may capture biologically meaningful features that extend beyond static mutation status. These properties help explain why exosomes are now viewed as promising substrates for cancer detection, classification, monitoring, and treatment-response evaluation [4,5,6,7,8,9].

The present review adds to this translational narrative by showing that exosomal cargo may be informative at several analytical scales. At the broadest level, pan-cancer exosome profiles can reveal recurring oncologic themes. At an intermediate level, disease-group comparisons can distinguish hematologic from solid malignancies. At a more refined level, tumor-type comparisons among individual solid cancers suggest that exosome profiling may assist with tissue-aware classification. This scale-dependent logic is one of the most important translational contributions of the present review [4,5,6,7,8,9,11,12,13,14,15,16,17,18,19,40,41,42,43,44,45,46,47,48].

Exosome-based biomarker development may also be strengthened by the physical properties of vesicles themselves. Encapsulation can protect proteins and RNAs from immediate degradation, thereby preserving biologically informative molecular features in body fluids. This relative stability is one reason why extracellular vesicles remain attractive candidates for liquid biopsy even in analytically challenging clinical contexts [4,5,6,7,8,9].

For this reason, the biomarker potential of exosomes is best framed in disciplined rather than promotional terms. Exosomes are promising not because they solve every problem of liquid biopsy, but because they occupy a biologically privileged position between tumor-state representation and active intercellular communication. Their real value will emerge only if clinical studies are designed with adequate standardization, biological context, and comparative rigor [2,4,5,6,7,8,9,10]. A further priority for clinical translation is to relate exosomal miRNA and target-gene identities to tumor stage and to patient survival. The curated presence/identity data analyzed here do not carry harmonized, patient-level clinical metadata (stage, quantitative expression, or mortality), so such correlations cannot be derived from the repositories themselves without unsupported assumptions. The appropriate route is to integrate the exosomal miRNA identities catalogued in this review (Supplementary Table S6) with stage- and survival-annotated resources such as TCGA and GEO expression cohorts, and ultimately with prospective plasma or urine exosome studies that include matched staging and follow-up, so that candidate onco-miRs can be tested for stage-dependent expression and prognostic value.

## 13. Methodological Considerations, Limitations, and Sources of Bias

A scientifically rigorous review of cancer exosome datasets must confront methodological heterogeneity directly. Differences in isolation approach, sample source, pre-analytical handling, storage, vesicle enrichment method, and downstream analytical platform can profoundly alter the apparent content of exosomal preparations. For proteins, mass spectrometry depth, database choice, peptide filtering, and protein inference rules can change the size and composition of the reported cargo set. For RNAs, extraction chemistry, sequencing platform, read-processing strategy, normalization, and annotation database selection can have equally strong effects. Therefore, cross-cancer comparisons are only as robust as the comparability of the underlying workflows. Although an integrative framework is applied here, comparison of enrichment outputs does not eliminate this underlying source heterogeneity [2,4,5,7,8,9,10].

The non-uniform size of the datasets included in the figures represents a second limitation. Some cancer types are represented by very large inventories, whereas others contribute relatively modest numbers of proteins or miRNAs. Large datasets are not inherently more correct than small datasets; however, they are more likely to generate broader enrichment patterns and more stable-looking functional architectures. Without careful interpretation, this can create an illusion of stronger biological coherence in the best-sampled disease categories [4,7,8,10].

A third issue involves the interpretive limits of enrichment analysis itself. GO and KEGG are powerful organizational tools, but they do not measure pathway activity directly. Enrichment indicates structured association within a dataset, not causal dominance in vivo. This distinction is especially important in exosome research, where vesicle preparations may include mixed subpopulations and where predicted miRNA targets may not be functionally engaged in recipient cells. Consequently, enrichment analysis should be treated as a scaffold for reasoning rather than a substitute for mechanistic experimentation [3,4,5,6,7,8,9,10].

Finally, publication bias and disease-selection bias must also be acknowledged. Tumor types with more active exosome research communities, easier access to clinically relevant body fluids, or stronger biomarker motivation are more likely to generate dense datasets and repeated validation attempts. This unevenness affects not only how much information is available, but also which biological narratives become dominant in the literature [4,5,6,7,8,9]. A further limitation concerns the specificity of exosome-based liquid biopsy in the presence of normal-cell background. Circulating exosomes in any biofluid are a composite of tumor, stromal, immune, and normal epithelial contributions, and this dilution of the tumor-derived fraction is the principal factor limiting the specificity of exosome-based diagnostics. Abundance in a repository does not by itself establish tumor origin. Strategies used in the field to mitigate this include immuno-affinity enrichment against tumor- or tissue-associated surface markers, selection of cargo showing high tumor-versus-normal differential abundance, and orthogonal confirmation of candidate markers in matched normal-donor samples. Accordingly, the liquid-biopsy potential described in this review should be regarded as conditional on adequate tumor-signal enrichment rather than as an intrinsic property of exosomes. Related to this, body-fluid provenance is not uniformly annotated across the source repositories. This is most consequential for the urologic cancers (kidney and prostate), whose entries likely combine urinary and systemic exosomes; the tissue-of-origin specificity of these categories is therefore limited, and fully resolving it would require primary studies with harmonized, single-biofluid sampling. Finally, the consensus miRNA-target rule adopted in Section 2.5 is specificity-weighted and may under-represent genuine interactions of low-abundance miRNAs, which should be considered when interpreting the miRNA-derived functional patterns.

## 14. Conclusions

Cancer-derived exosomes occupy a unique position in oncology because they are simultaneously messengers, mirrors, and potential biomarkers of malignant disease. The integrated framework developed in this review indicates that exosomal proteins and microRNAs from multiple cancer types can be organized into coherent functional patterns and compared across several analytical scales. Pan-cancer analysis highlights shared biological themes related to signaling, transport, extracellular interaction, and regulatory communication. Disease-group comparisons between hematologic and solid malignancies suggest that exosomal cargo reflects broader pathological ecology. Together, these findings support the view that exosomes are not passive byproducts of secretion, but structured and biologically informative components of tumor communication [1,3,4,5,6,7,8,9,11,12,13,14,15,16,17,18,19,40,41,42,43,44,45,46,47,48]. The specific value added by this unified re-analysis, beyond the annotation of any individual dataset, is the identification of these cross-cancer regularities—a conserved, lineage-independent functional core together with reproducible disease-group-specific signatures—which emerge only when all eight cancers are analyzed under one identical framework.

At the same time, the review reinforces an equally important conclusion: biological promise does not yet equal clinical readiness. Additional validation, dataset harmonization, standardized reporting, and mechanistic follow-up remain essential before exosome-based profiling can be translated reliably into precision oncology. Nevertheless, the combined analysis of protein and microRNA cargo provides a strong conceptual basis for future studies in cancer detection, disease stratification, monitoring, and biomarker-driven translational research [2,3,4,5,6,7,8,9,10].

## 15. Future Directions

Future progress in cancer exosome research will depend on moving from descriptive cataloging to analytically disciplined and clinically contextualized validation. One immediate priority is standardization. The field needs better alignment in sample handling, vesicle isolation, reporting practices, and bioinformatic annotation so that cross-study comparisons become more interpretable and more reproducible [2,4,5,7,8,9,10].

A particularly promising direction is the development of context-aware multi-omic signatures. Instead of searching for one universal exosome marker, future studies should evaluate how proteins, miRNAs, and other vesicle-associated analytes can be integrated into composite signatures tailored to specific clinical questions. The optimal signature for detecting recurrence in a hematologic malignancy may differ fundamentally from that needed to stratify risk in a solid tumor or to distinguish between related epithelial cancers [3,4,5,6,7,8,9,11,12,13,14,15,16,17,18,19,40,41,42,43,44,45,46,47,48].

Equally important is the need to connect exosome profiling more directly to mechanism. If a vesicle-associated protein or miRNA is proposed as biologically meaningful, future studies should test whether it measurably alters recipient-cell behavior, therapy response, or metastatic adaptation. The most clinically useful exosome biomarkers are likely to emerge when descriptive profiling is tied to experimentally grounded biology [1,3,4,5,6,7,8,9].

## Data Availability

The datasets analyzed in this study, including the input protein and miRNA lists and all enrichment output tables, are provided in the Supplementary Materials.

## Supplementary Materials

The following supporting information can be downloaded at https://www.mdpi.com/article/10.3390/ijms27157057/s1.

## Abbreviations

The following abbreviations are used in this manuscript:

EVs	Extracellular vesicles
GO	Gene Ontology
KEGG	Kyoto Encyclopedia of Genes and Genomes
PANTHER	Protein ANalysis THrough Evolutionary Relationships
HGNC	HUGO Gene Nomenclature Committee
TME	Tumor microenvironment
ECM	Extracellular matrix
BP	Biological process
CC	Cellular component
MF	Molecular function
miRNA	MicroRNA
mRNA	Messenger RNA
ESCRT	Endosomal sorting complex required for transport
EMT	epithelial-to-mesenchymal transition
MVB	multivesicular body
ILV	intraluminal vesicle
UniProt	Universal Protein Resource
miRTarBase	miRNA Target database
